# Species‐level image classification with convolutional neural network enables insect identification from habitus images

**DOI:** 10.1002/ece3.5921

**Published:** 2019-12-24

**Authors:** Oskar L. P. Hansen, Jens‐Christian Svenning, Kent Olsen, Steen Dupont, Beulah H. Garner, Alexandros Iosifidis, Benjamin W. Price, Toke T. Høye

**Affiliations:** ^1^ Department of Bioscience Center for Biodiversity Dynamics in a Changing World (BIOCHANGE) Aarhus University Aarhus C Denmark; ^2^ Department of Bioscience Section for Ecoinformatics and Biodiversity Aarhus University Aarhus C Denmark; ^3^ Natural History Museum Aarhus Aarhus C Denmark; ^4^ Life Sciences Natural History Museum London UK; ^5^ Department of Engineering ‐ Signal Processing Aarhus University Aarhus N Denmark; ^6^ Department of Bioscience and Arctic Research Centre Aarhus University Rønde Denmark

**Keywords:** arthropod sampling, automatic species identification, camera trap, entomological collection, image classification, image database

## Abstract

1. Changes in insect biomass, abundance, and diversity are challenging to track at sufficient spatial, temporal, and taxonomic resolution. Camera traps can capture habitus images of ground‐dwelling insects. However, currently sampling involves manually detecting and identifying specimens. Here, we test whether a convolutional neural network (CNN) can classify habitus images of ground beetles to species level, and estimate how correct classification relates to body size, number of species inside genera, and species identity.
2. We created an image database of 65,841 museum specimens comprising 361 carabid beetle species from the British Isles and fine‐tuned the parameters of a pretrained CNN from a training dataset. By summing up class confidence values within genus, tribe, and subfamily and setting a confidence threshold, we trade‐off between classification accuracy, precision, and recall and taxonomic resolution.
3. The CNN classified 51.9% of 19,164 test images correctly to species level and 74.9% to genus level. Average classification recall on species level was 50.7%. Applying a threshold of 0.5 increased the average classification recall to 74.6% at the expense of taxonomic resolution. Higher top value from the output layer and larger sized species were more often classified correctly, as were images of species in genera with few species.
4. Fine‐tuning enabled us to classify images with a high mean recall for the whole test dataset to species or higher taxonomic levels, however, with high variability. This indicates that some species are more difficult to identify because of properties such as their body size or the number of related species.
5. Together, species‐level image classification of arthropods from museum collections and ecological monitoring can substantially increase the amount of occurrence data that can feasibly be collected. These tools thus provide new opportunities in understanding and predicting ecological responses to environmental change.

## Graphical abstract

Species identity can be inferred from simple images of an extensive and highly accurate carabid beetle collection. Such image classification will be instrumental in efforts to increase the rate at which crucial occurrence data for insects are generated in the future.

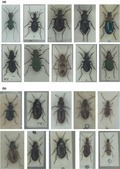

## 1 INTRODUCTION

Recent reports suggest that insect biomass and abundance have been declining dramatically in recent decades (Agrawal & Inamine, 2018; Hallmann et al., 2017; Lister & Garcia, 2018; Loboda, Savage, Buddle, Schmidt, & Høye, 2018; Seibold et al., 2019; Wagner, 2019), even though trends vary if measured across or on individual habitats and species (Loboda et al., 2018). Estimating and tracking changes in abundance and diversity of insects at species level through time and space is critical to understand the underlying drivers of change and to devise possible mitigation strategies. Methods that enable error estimation in observations, with high data quantity, quality, and resolution on spatial, temporal and taxonomic scales are crucial.

To date, no efficient method enables tracking of insect activity, abundance, and diversity in a nondestructive, cost‐effective, and standardized way. Common sampling methods including direct observations, a variety of trapping methods, direct sampling methods, and DNA‐based methods all fail on one or two of these criteria. A much criticized but widely used method is pitfall traps (Brown & Matthews, 2016; Engel et al., 2017; Skvarla, Larson, & Dowling, 2014). Like other trapping methods such as malaise traps and pan traps, they remove study specimens from the environment, thus being invasive. Furthermore, each trapping method comes with its own set of biases or methodological idiosyncratic behaviors, making interpretations across habitats difficult (Skvarla et al., 2014). Given the sampling method and in order to increase the number of individuals trapped this often comes at the expense of coarse temporal information (several days or weeks; Schirmel, Lenze, Katzmann, & Buchholz, 2010). The resulting low temporal resolution in activity estimate defined by the sampling frequency can only be related to environmental factors over the same time scale (Asmus et al., 2018; Høye & Forchhammer, 2008). Direct observations, being nondestructive, currently require identification of organisms by trained ecologists or taxonomists at the study site throughout the sampling period, greatly reducing the number of feasible samples.

The camera trap method has distinct advantages over traditional methods in entomology. Compared to the often used pitfall traps, camera traps sample more individuals (Collett & Fisher, 2017; Halsall & Wratten, 1988), and cause no depletion of specimens or habitat destruction (Digweed, Currie, Carcamo, & Spence, 1995; Zaller et al., 2015). Furthermore, camera traps require less maintenance (Caravaggi et al., 2017; Collett & Fisher, 2017). The average movement speed and various behavioral traits of a species can be directly measured between single frames of one camera trap (Caravaggi et al., 2017), allowing true abundance of species to be estimated based on their movement speed and range. Rarely, but increasingly, camera traps have been used to monitor insects and other arthropods (Collett & Fisher, 2017; Dolek & Georgi, 2017; Zaller et al., 2015). Even though identifications of species based on images are well known for mammals and birds (Norouzzadeh et al., 2018; Yu et al., 2013), camera trap studies designed for arthropods conclude that image‐based species identification by humans is generally not possible (Collett & Fisher, 2017; Zaller et al., 2015).

Image‐based species identification methods on arthropods have been applied with success on samples in the laboratory (Joutsijoki et al., 2014). In order to fully implement the advantages of camera traps, there is a need for implementing image classification techniques to automatically identify and recognize species (Weinstein, 2017). Deep convolutional neural networks have together with the release of machine learning frameworks like TensorFlow (Abadi et al., 2015) and available models like Inception or GoogleNet (Szegedy et al., 2015; Szegedy, Vanhoucke, Ioffe, Shlens, & Wojna, 2016) have advanced significantly in recent years (Wäldchen & Mäder, 2018). Image classifications used for species identification have dramatically increased in accuracy, performance, and in the number of taxa analyzed (Marques et al., 2018; Martineau et al., 2017; Norouzzadeh et al., 2018; Schneider, Taylor, & Kremer, 2018; Van Horn et al., 2017). On a limited number of species, identification by computers can be as good as human experts and with less variation in accuracy (Ärje et al., 2019). Automated species identification has also been successfully implemented on the citizen science portal iNaturalist.org, enabling a suggested list of species for an observation, based on the existing archive of image data (Van Horn et al., 2017).

We test the ability of a convolutional neural network (CNN) to classify ground beetles (Coleoptera: Carabidae) to genus, species, or higher taxonomic level from images of specimens within the British collection at the Natural History Museum, London. This collection provides a good test case as it has been well curated and assessed for correct species identity, represents a commonly prepared type of insect collection for which this method is directly applicable to, and has access to the SatScan^®^ (SmartDrive Limited; Blagoderov, Kitching, Livermore, Simonsen, & Smith, 2012; Mantle, LaSalle, & Fisher, 2012), a rapid whole drawer imaging system. Beetle specimens are placed in unit trays inside drawers, prepared either glued onto card or pinned and are generally positioned in dorsal view with head in the same direction, reducing the variability in the data. These prepared specimens can serve a simplified model for what a camera trap would record. Thus, these images represent a good indicator of the potential taxonomic resolution of automatic species identification with current state of the art classification methods, based on data from a camera trap, when compared to expert identifications of the specimens. Specifically, we quantify the number of correct species identifications of carabid beetles based on image classification of habitus images. Furthermore, we assess variation in correctly classified images among taxa. In particular, we test how classification recall (number of images classified to a group from the total number of images within the group) varies among genera and for specimens of different body size. To increase accuracy and to critically assess reliability, we postprocess the output and apply thresholds on confidence values for each of the included taxonomic levels to avoid low confidence in predictions.

## 2 MATERIALS AND METHODS

### 2.1 Obtaining images

**Figure 1:**
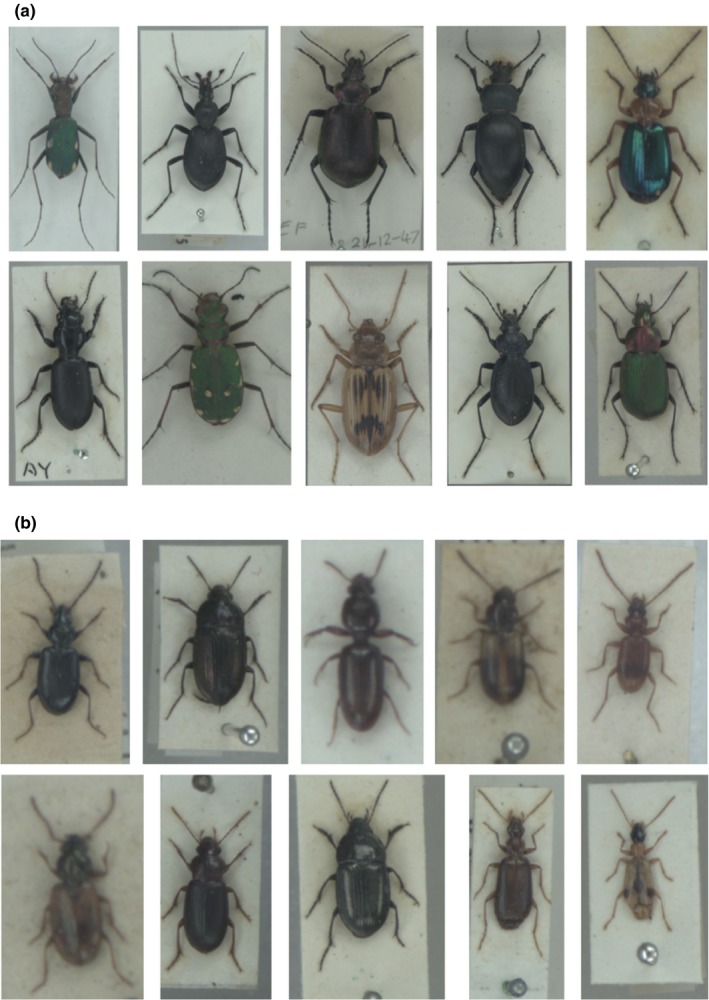
Specimens used to train or test the convolutional neural network. (a) Species with accuracy (images of species in test dataset classified to correct species) on 90% or more. First row: *Cylindera germanica*, *Cychrus caraboides*, *Calosoma inquisitor*, *Carabus glabratus*, and *Lebia chlorocephala.* Second row: *Broscus cephalotes*, *Cicindela campestris*, *Nebria complanata*, *Carabus problematicus*, and *Chlaenius nigricornis*. (b) Species drawn randomly from the remaining 281 species. First row: *Bembidion atrocaeruleum*, *Amara famelica*, *Dyschirius politus*, *Acupalpus meridianus*, and *Blemus discus.* Second row: *Bembidion ephippium*, *Ophonus melletii*, *Amara lunicollis*, *Dromius angustus*, and *Demetrias imperialis*

In August 2017, we scanned the British collection of ground beetles (Coleoptera: Carabidae) at the Natural History Museum London using the SatScan^®^ (Blagoderov et al., 2012; Mantle et al., 2012). The collection comprised 207 drawers with specimens curated and identified to species level (Figure 1). All drawers were scanned with the same light and exposure settings following the imaging protocol described by Blagoderov et al. (2012) and resulted in images of 15,828 × 15,565 pixels (36 pixels/mm) per drawer.

Drawer images were segmented into specimens using Inselect version 0.1.36 (Hudson et al., 2015) followed by manual refinement by two people, resulting in 65,841 single‐specimen images (per species mean = 182, range = 1–892). To reduce variability and avoid images of exceptional preparations, specimens mounted with dorsal side facing down, or with head, pronotum, or elytra missing, and larvae were tagged during the manual quality check and refinement step. Each specimen image was also tagged with the taxon name (genus and species), according to the collection data (361 taxa). We excluded specimens without a taxon name (66 specimens) or without proper identification to species level (100 specimens), larvae (27 specimens) and specimens mounted with dorsal side downward (296 specimens) or missing either the head, pronotum, or elytra (504 specimens). In order to secure sufficient image data to test the classification success, only species with 50 specimens or more were included, thus excluding additional 70 species and 1,550 specimens. The taxonomic classification used for the species was from gbif.org via the *taxize* R‐package (Chamberlain & Szöcs, 2013). Afterward additional taxonomic levels were added such as family, subfamily, tribe, subgenus, and the ordered taxonomic hierarchy from the British checklist of beetles (Duff, Lott, Buckland, & Buckland, 2012).

### 2.2 Training and testing the convolutional neural network

The complete dataset comprised 63,364 specimen images from 291 species (images per species mean = 218, range = 50–888; Figure 1) comprising 80 genera. For each species, specimen images were divided into three groups for training (50%), validating (20%), and testing (30%) the network, respectively. In order to assign images consistently to the three datasets, we generated a probability value for each image based on the output from encrypting the filename. Images with percentage 0–20 were assigned as validation, 20–50 as testing and above 50 as training. Thus, the division percentages did not entirely reflect the number of images in each of the datasets with 31,533 (49.8%), 25,334 (20.0%), and 19,164 (30.2%) images used for training, validation, and testing, respectively. While the training and validation images were used only for training of the model, the test images, not known by the retrained model, were used for further analysis. We used the scripts developed by TensorFlow (Abadi et al., 2015) for training an Inception‐v3 model (Szegedy et al., 2016) initially trained on ImageNet database (Deng et al., 2009), following the tutorial from Tensorflow (Tensorflow, 2019). The retraining was run in TensorFlow version 1.13.1, python version 3.7.3. Input images were resized to 299 × 299 pixels regardless of input image size and shape to follow the model specification. The model was trained with gradient decent optimizer for 225,000 iterations and a batch size of 100 for both training and validation datasets to reach at least 700 epochs. We did not apply augmentation of images; however, we tested that the learning rate of the model was optimized by training the model with learning rates of 0.5, 0.3, 0.1, 0.045, 0.01, 0.001, and 0.0001. We choose the default learning rate, that is, 0.045, which produced similar validation accuracy as 0.5, 0.3, and 0.1 with lower learning rate. Thus choosing the hyperparameter with smallest optimization update while the validation accuracy converged during training steps. The output layer of the CNN, activated by a softmax function, gave a predicted confidence value for each of the 291 species in each image ranging between 0 and 1.

### 2.3 Evaluating predictions and setting thresholds to separate low‐ and high‐confidence predictions

The output layer of the convolutional neural network consisted of a vector with a confidence value for each class (i.e., species) included in the neural network. The class with the highest value (top 1 or top 5) from the output layer was interpreted as the predicted class for an image. We assessed if the image was predicted correctly, if the ground truth name appeared in top 1 or, in a separate measure, in top 5.

For each species we calculated from all test images, true positives (tp): ground truth and predicted as ground truth, false positives (fp): not ground truth and predicted as ground truth, true negative (tn): not ground truth and not predicted as ground truth, false negative (fn): ground truth and not predicted as ground truth. Based on these numbers, we evaluated classification precision: tp/(tp + fp), classification recall: tp/tp + tn), classification accuracy: (tp + tn)/(tp + tn + fp + fn), True positive rate (TPR): tp/(tp + fn), true negative rate (TNR): tn/(tn + fp), and balanced classification accuracy: (TPR + TNR)/2.

The neural network included only species‐level classes. To assess the number of correctly classified images on levels above the species level, we calculated a new set of confidence values through the sum of all classes in the higher taxonomic level (e.g., the confidence value sum of all species belonging to the same genus). We repeated this procedure for all taxonomic levels (subgenus, genus, tribe, subfamily, and family).

We introduced a minimum confidence value threshold to assess at which taxonomic resolution an image could be classified. Starting at species‐level resolution, we evaluated if the highest confidence value was below the threshold value. If the highest confidence value was lower than the threshold value, we repeated the evaluation for classes at the next taxonomic level, that is, at lower taxonomic resolution.

### 2.4 Analysis

In total 19,164 images of 291 species (mean images per species = 65.9, range = 11–272) were used as test images, not involved in the training and validation. As the number of images was not equal for all species, classification recall was calculated for each species as the proportion of images correctly classified. We used two generalized linear models with binomial distribution to assess if a classification of an image was correct or not. In model 1, only species identity was used as explanatory variable. In model 2, we used image size measured in megapixels extracted from the image metadata (exif) using *exiftool* v.11.06 through *exifr* r‐package (Dunnington & Harvey, 2019) as a measure of body size (hereafter referred to as body size), the number of species within its genus, and the top 1 value from the output layer in the convolutional neural network as explanatory variables. A sensitivity analysis was performed for model 2, to separate effects from the three explanatory variables on the prediction. This analysis kept all but one variable constant at mean value for number of training images, body size, and top1 value or median for number of species inside genus.

## 3 RESULTS

Of the 19,164 test images, 9,949 (51.9%) were predicted to the correct species (threshold = 0, n species = 291), while when extracting genus names of predictions and ground truths 14,357 (74.9%) were predicted to the correct genus (n genera = 80). For predictions on species level, mean classification precision, recall, accuracy, and balanced accuracy were 54.7%, 50.7%, 99.7%, and 75.3%, respectively (Table S1).

The confusion matrix, based on all species‐level predictions, revealed that species were often confused with other species within the same genus (Figure S1). Some typical confusions were also between tribes: Bembidiini species were often mistaken as Lebiini species and vice versa. On the other hand, Bembidiini were rarely mistaken as Zabrini, while Zabrini were mistaken as Bembidiini in more images. On the subfamily level, two tiger beetles (Cicindelinae) were misidentified as belonging to one of the other subfamilies, while six species of Carabinae had at least one image predicted as Cicindelinae (Figure S1).

By excluding images with low confidence (<0.25) on any one specific species (top 1 value), 56.6% and 77.8% of a total of 16,812 images were predicted to the correct species and genus, respectively. The resulting mean classification precision, recall, accuracy, and balanced accuracy were 58.2%, 54.0%, 99.7%, and 76.9%, respectively. Average recall per species was 50.7% (min 10.6, max 100%, *SD* 20.8%, *SE* 1.2%; Figure S2).

**Figure 2:**
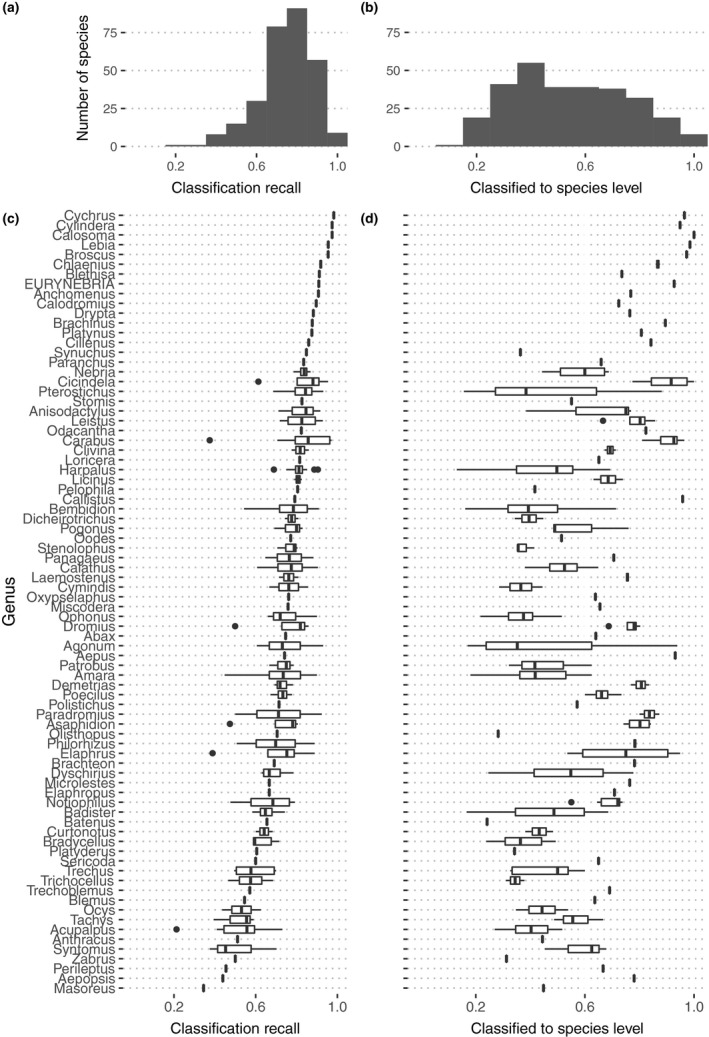
Classification performance, when setting minimum acceptable confidence threshold to 0.5. (a) Distribution and (c) genus‐summary of classification recall (i.e., proportion of images of a species classified to correct taxon regardless of the predicted taxonomic level, e.g., to species, genus). (b) Distribution and (d) genus‐summary of images classified to species level (i.e., proportion of images of a species classified to species level), as an indicator of classification taxonomic resolution. A large proportion of images identified to species level indicate a high taxonomic resolution, while the taxonomic resolution gradually decreases when larger proportions are identified correctly only to higher taxonomic levels (e.g., genus, tribe, or subfamily)

Setting a minimum acceptable confidence threshold to 0.5 before decreasing taxonomic resolution by one hierarchical level (i.e., summing all species‐level confidence values from species belonging to that group e.g., all species in a genus), 75.8% of a total of 19,164 images were classified correctly to the decided taxonomic level and average classification recall across all specimens increased to 74.6% (min 21.3%, max 98.2%, *SD* 13.2%, *SE* 0.8%). Mean balanced accuracy, precision, and recall varied with taxonomic resolution (Table 1). Classification recall and taxonomic resolution varied considerably among the 291 species and 80 genera (Figure 2). For most species, the proportion of images correctly classified was above 76.9% (median, Figure 2a). Of the 14,527 correctly classified images, 7,362 were correct at the species level, while the remaining 7,155 were classified correctly with less taxonomic resolution than species level. Most species had some images predicted with a varying taxonomic resolution. Very few species had all images classified to a specific taxonomic resolution level (Figure 2b).

**Table 1:** Number of specimens at each taxonomic resolution, mean balanced accuracy, mean precision, and mean recall when setting a minimum acceptable confidence threshold to 0.5 before decreasing taxonomic resolution

Taxon rank	No. specimens	Mean balanced accuracy	Mean precision	Mean recall
Species	10,348	82.4	70.0	64.9
Subgenus	1,799	73.1	75.3	46.5
Genus	3,212	65.0	72.3	30.6
Tribe	1,231	64.1	72.0	30.1
Subfamily	2,573	70.0	66.6	66.6
Family	1	NA	100	100
Weighted mean	19,164	75.8	70.6	55.4

Note

Bottom row gives the mean of measures weighted by the number of specimens at each taxon rank.

Without setting a threshold, ten species had a classification recall of 90.0% or greater (Table 2; Figure 1a). With the 0.5 confidence threshold, six species had a classification recall of 90.0% or greater; however, the number of false predictions (false positives) was reduced for all ten species (i.e., increasing the recall; Table 2). With the 0.5 confidence threshold, 27 species had more than 85.0% of their test images classified at the species level, while seven species had less than 20.0% images classified at the species level (Figure S3). Genera with many species and including species which are traditionally hard to identify such as Bembidion, Agonum, Amara, Harpalus, and Pterostichus had a mean proportion of images classified to species level per species in the range 41.7%–45.5% (Figure S3). Inside these genera, the mean minimum and mean maximum proportion of test images classified to species level was 16.0% and 77.0%, indicating a high variability inside some genera (Figure S3).

**Table 2:** Species with 90% or more of specimens predicted to correct species if no threshold was set

Species	Number of specimens	No threshold		Threshold 0.5		
		True positive (%)	False positive	True positive (%)	Not species level	False positive
*Cylindera germanica*	39	100	12	94.9	2	4
*Cychrus caraboides*	57	98.2	13	96.5	2	4
*Calosoma inquisitor*	39	97.4	8	97.4	0	3
*Carabus glabratus*	27	96.3	2	88.9	2	NA
*Lebia chlorocephala*	67	95.5	3	94.0	1	3
*Broscus cephalotes*	111	93.7	14	92.8	3	4
*Cicindela campestris*	88	93.2	NA	92.0	3	NA
*Nebria complanata*	55	92.7	1	89.1	4	NA
*Carabus problematicus*	107	91.6	16	87.9	7	14
*Chlaenius nigricornis*	40	90.0	4	82.5	5	3

Note

Number of specimens in test dataset, percentage of specimens predicted to the correct species, and false positives (i.e., the number of specimens predicted to the wrong species). For threshold 0.5, the percentage of specimens in species predicted to the correct species, the number of specimens that did not meet the threshold, thus not predicted on species level, and false positives (i.e., the number of specimens predicted to the wrong species).

**Figure 3:**
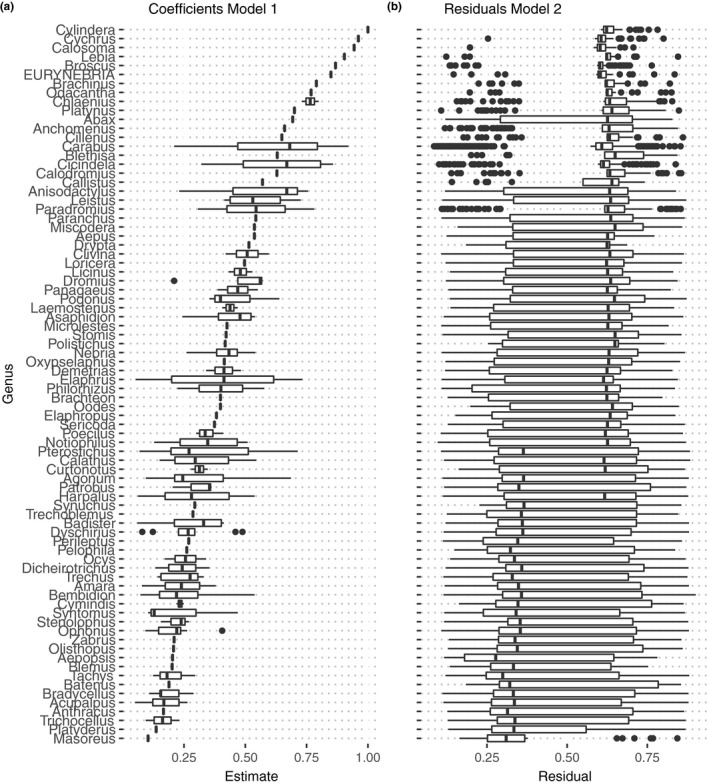
(a) Model coefficients from GLM model 1 (explanatory variable: species identity), representing species coefficients and (b) residuals from model 2 (explanatory variables: body size, top 1 value from last layer in convolutional neural network, number of species in genus, and number of training images) representing residuals for images. Species identity and number of species in genus were strongly linked, in order to keep species identity separate from other explanatory variables two models were used and residuals from model 2 compared with coefficients from model 1 compared on genus level. Higher residual values indicate that other explanatory variables than included in model 2, explain more of the variation

Images classified to correct species were explained by top 1 value from the last layer in the convolutional neural network, body size, number of species within the same genus, and species identity (Figure 3; Table 3; Figure S4). The number of species within the same genus had a negative relationship with the probability of classifying to correct species while body size and the top 1 value from the last layer in CNN were positively correlated with the probability of correctly classifying the species (Table 3; Figure S4). Species identity did affect the estimate of model 1, while residuals from model 2 covaried with the estimates, suggesting that explanatory variables not included in the model could be important.

**Table 3:** Model coefficients and 95% confidence interval from a generalized linear model (model 2), predicting if an image is classified to correct species by the convolutional neural network as binomial variable (true/false)

Parameter	Lower CI (2.5%)	Estimate	Upper CI (97.5%)
(Intercept)	0.0639	0.0713	0.0793
Top 1 value	0.986	0.988	0.990
Body size	0.616	0.659	0.701
Number of species	0.497	0.498	0.498
Number of training images	0.500	0.500	0.500

Note

Explanatory variables included the top1 value in the output layer, body size in megapixels, number of species in the same genus as the ground truth species, number of training images of the ground truth species.

## 4 DISCUSSION

Within the tested species of British Carabidae, 51.9% of the 19,164 images were classified to the correct species, when testing the model classifying to species level, and 74.9% to the correct genus, using the same trained model with genus names from ground truth and predicted species. However, the classification success for images varied significantly between species and genera, with species being everything from very difficult or very easy for the model to predict to species level. Specifically ten of 291 species had more than 90% of their images classified correctly at species level, without setting a threshold. When setting a threshold to 0.5 as minimum confidence value before decreasing taxonomic resolution, most species did, however, not reach taxonomic resolution at species level. The average classification recall increased from 50.7% to 75.8% using the threshold. A range of hyperparameters could have been optimized further, like learning rate and augmentation of images. This would likely have increased the overall classification recall. However, the general patterns in body size and number of species in genus would most likely remain the same. Body size of the specimens positively contributed to the models ability to classify an image. When setting a minimum threshold to the confidence level, more images were classified correctly; however, this came at the cost of losing taxonomic resolution in their prediction. In spite of the reduced taxonomic resolution, such a model can prove extremely useful in applied situations where no taxonomic information is attached beforehand, for example, reducing workload of counting, classifying on broader taxonomic levels, and creating an overview of a collection being received by a museum.

Modifying the top layer of the CNN based on the images that we extracted from the collection enabled us to distinguish among 291 classes. That is most of all species known to occur in the British Isles within ground beetles, a family belonging to one of the most species‐rich orders of animals, Coleoptera with 380,000 described species (Zhang, 2011). As in other taxonomic groups, carabid beetles contain species that are morphologically only differentiated with subtle differences, which the result of this model reflected and handled to some extent by decreasing the taxonomic resolution on those image predictions, that is prediction to genus. Studies have used convolutional neural networks to classify species of a wide range of taxa, including arthropods and mammals (Norouzzadeh et al., 2018; Van Horn et al., 2017). However, this is the first to use a dataset within a well‐defined geographical and taxonomic species‐rich unit as well as providing information on how the postprocessing of the classification can trade‐off taxonomic resolution and classification recall. As all of the images in this dataset were taken with the same fixed camera settings and distance to object, the image size could be used as a proxy for body size. Larger specimens thus have more pixels in this dataset, which is the case when scanning drawers in collections and on camera traps faced toward a ground surface, using a camera with a fixed distance to the objects. Importantly, this also suggests that images from cameras only capture a limited body size range, as images with fewer pixels are less likely to be predicted to correct species.

Comparing our results to those obtained from other convolutional neural networks, built for the specific purpose of identifying other groups of arthropods (e.g., Marques et al., 2018), there is scope for increasing both classification recall and taxonomic resolution. Either image quality, network structure, number of classes to predict, or the image recording perspective could explain the differences. When comparing precision and accuracy of dorsal perspective images of ants by Marques et al. (2018) we achieve comparable results (precision 54.7% and balanced accuracy 75.3% vs. 52.0% and 59.0%). For a range of other studies that classify arthropods to species level, the results are comparable, even though fewer species are typically used in classification (Martineau et al., 2017). van Horn et al. (2017) presented a species level trained network based on Inception ResNet v2 and 675,000 images among 5,000 species of plants and animals. Their mean accuracy level within 1,021 insect species was 74.5%, which is comparable with our balanced accuracy of 75.3% of images in our study. When critically assessing confidence values and information of taxonomy, we increased the average accuracy above the level of van Horn et al. (2017) at species level, however, losing taxonomic resolution for nearly half of the images.

Automated or semi‐automated identification of insects on species or higher taxonomic levels has multiple potential applications, including in museum collections, ecological studies, and biodiversity monitoring. In museum collections, classification could identify specimens of accessions on entry into the museum and help taxonomic experts to find unusual specimens in the collections for focused taxonomic work. Classification of images enables cameras to be utilized as direct observers in ecological studies, providing detailed knowledge of species habitat preferences, activity levels, and species interactions. In monitoring, a continuous sampling of arthropod image data could also provide abilities to historically document and forecast abundances and activities of arthropods. However, all of the potential uses will only become achievable with considerable improvements of the accuracy as presented here. Proper testing and validation in applied contexts and in a broader range of taxa and habitats are crucial to achieve species‐level classification.

Even though we did not find a consistent error in all species, the results indicate that CNN can be used for a variety of classification tasks with high accuracy and for some species, high taxonomic resolution. Importantly, the results indicate that habitus images are sufficient to classify images to species level, albeit not for all species. Taxonomic classification based on habitus images is needed for camera trap‐based studies, where detailed images are not available. We show that assessing whether there is sufficient evidence to predict a specimen to a certain taxonomic resolution can be informed by the classification model output, through setting a confidence value threshold. Data from camera traps are possibly more complex and images from camera traps also need detection of objects, as multiple individuals may occur in the same frame. Object detection in camera traps has already been utilized for large mammals (Schneider et al., 2018), suggesting that object detection with CNN can be suitable for arthropods as well.

With the ability, from habitus images, to classify and know the classification error among arthropods including ground beetles, convolutional neural networks provide a practical tool. For ecologists, conservationists, and museum curators applied species‐level classification on massive datasets can provide new opportunities for predicting the consequences of environmental changes for living organisms.

## CONFLICT OF INTEREST

None declared.

## AUTHOR CONTRIBUTIONS

OLPH, TTH, KO, and JCS conceived the ideas; OLPH, TTH, KO, JCS, and AI designed the methodology; OLPH, BP, BG, and SD collected the data; OLPH analyzed the data; OLPH led the writing of the manuscript. All authors contributed critically to the drafts and gave final approval for publication.

## Data Availability

A total of 63,364 images in folders corresponding to species will be released at zenodo.org under creative commons license attribution 4.0 International (https://doi.org/10.5281/zenodo.3549369).
