# Supplementary material for: Species‐level image classification with convolutional neural network enables insect identification from habitus images
Source: Ecol Evol. 2019 Dec 24;10(2):737–47. doi: 10.1002/ece3.5921 (PMC6988528; doi:10.1002/ece3.5921)
Supplement: Supplementary file 1 [file ECE3-10-737-s001.docx]

# Supporting Information


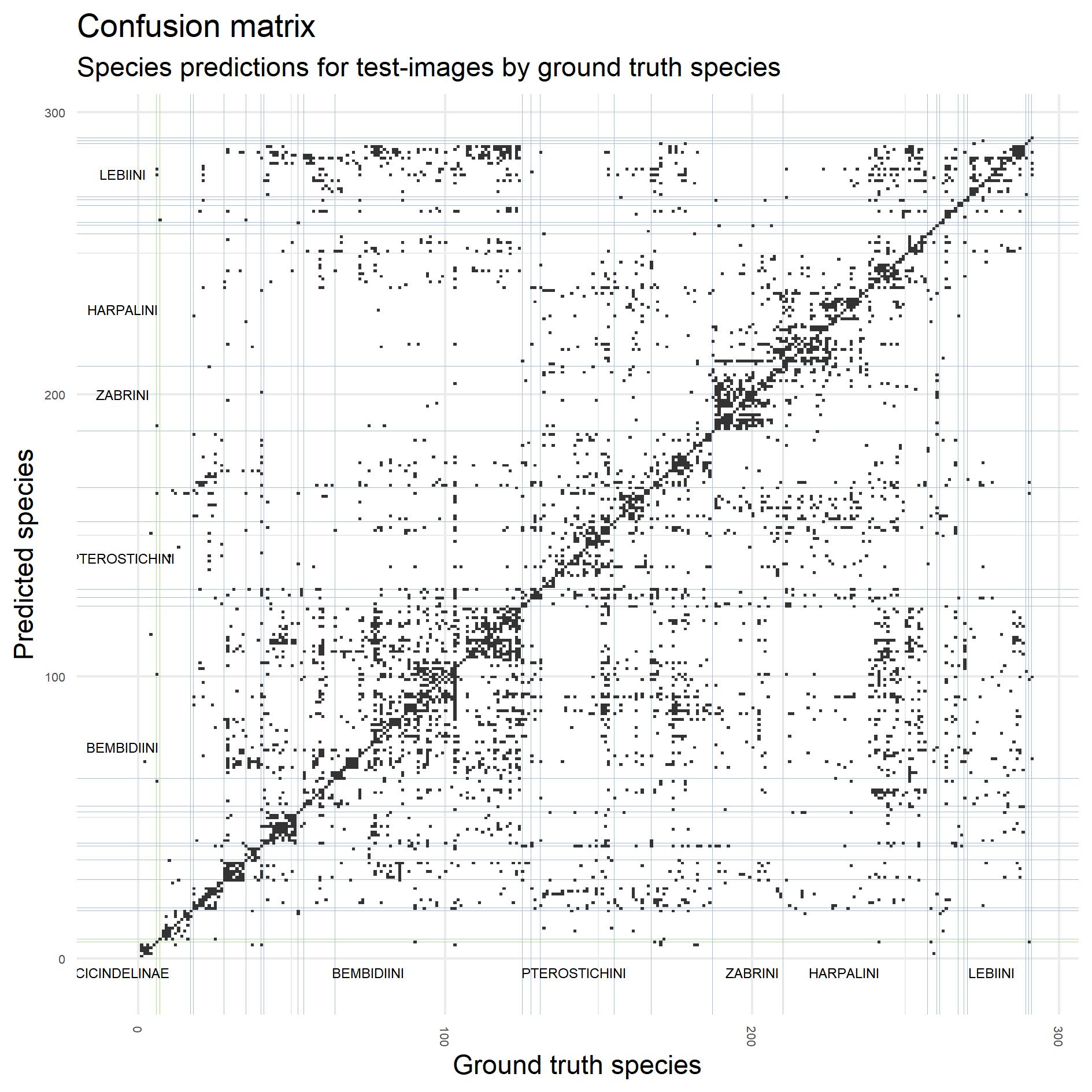


**Figure S1:** Confusion matrix for all 291 classes (species) from last layer of the convolutional neural network. Squares represent the outcome of image predictions of a ground truth species. Lines denote the first species in subfamilies (green) and tribes (blue).


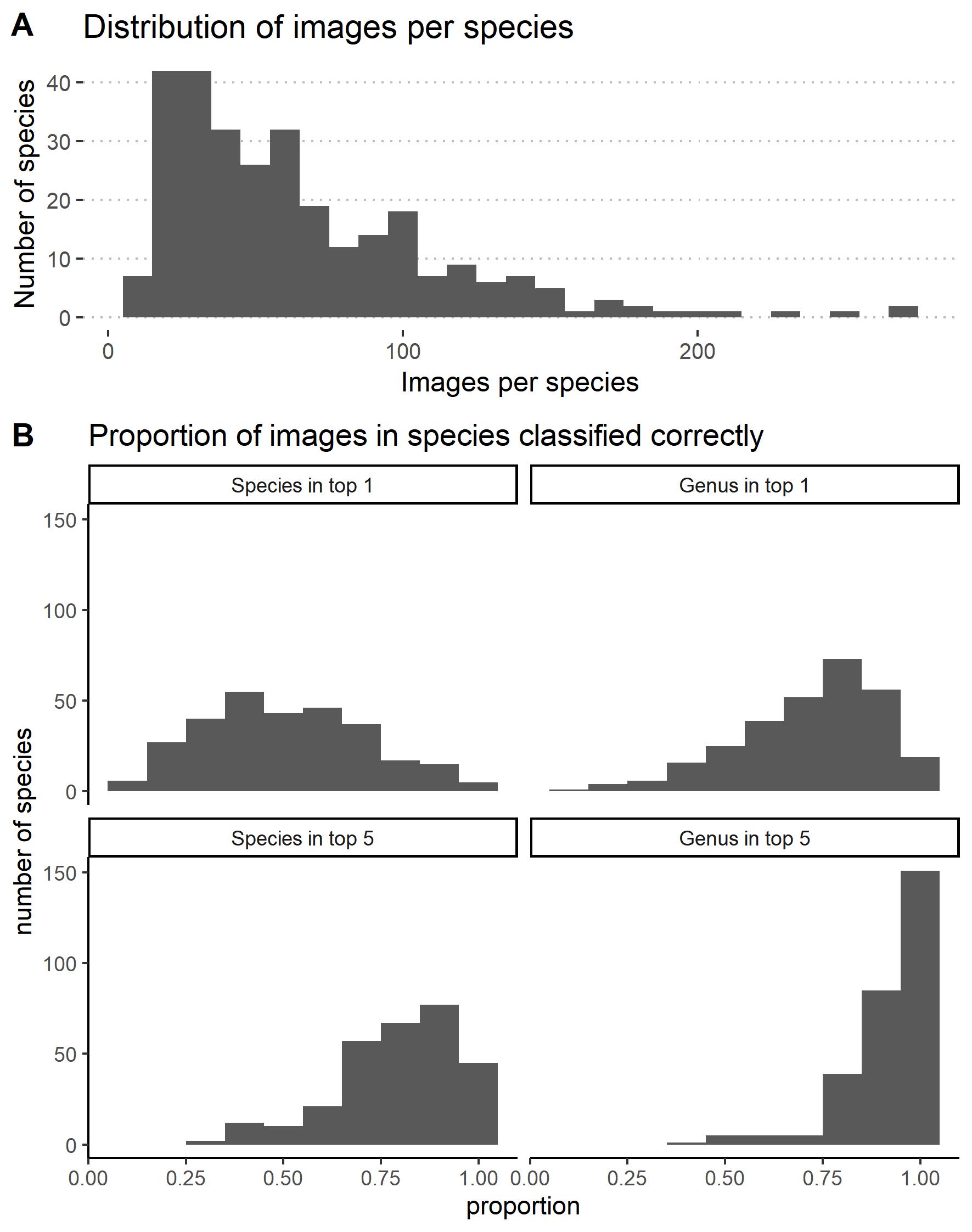


**Figure S2:** Images in test-dataset, including A: Number of Images per species. B: Proportion of images classified correctly per species for four different criteria of being considered correct: top1 predicted species must be ground truth species, genus belonging to top1 predicted species must be the same as ground truth species, species in top 5 predicted species must contain ground truth species, or genera in top5 predicted species must contain the genus belonging to ground truth species.


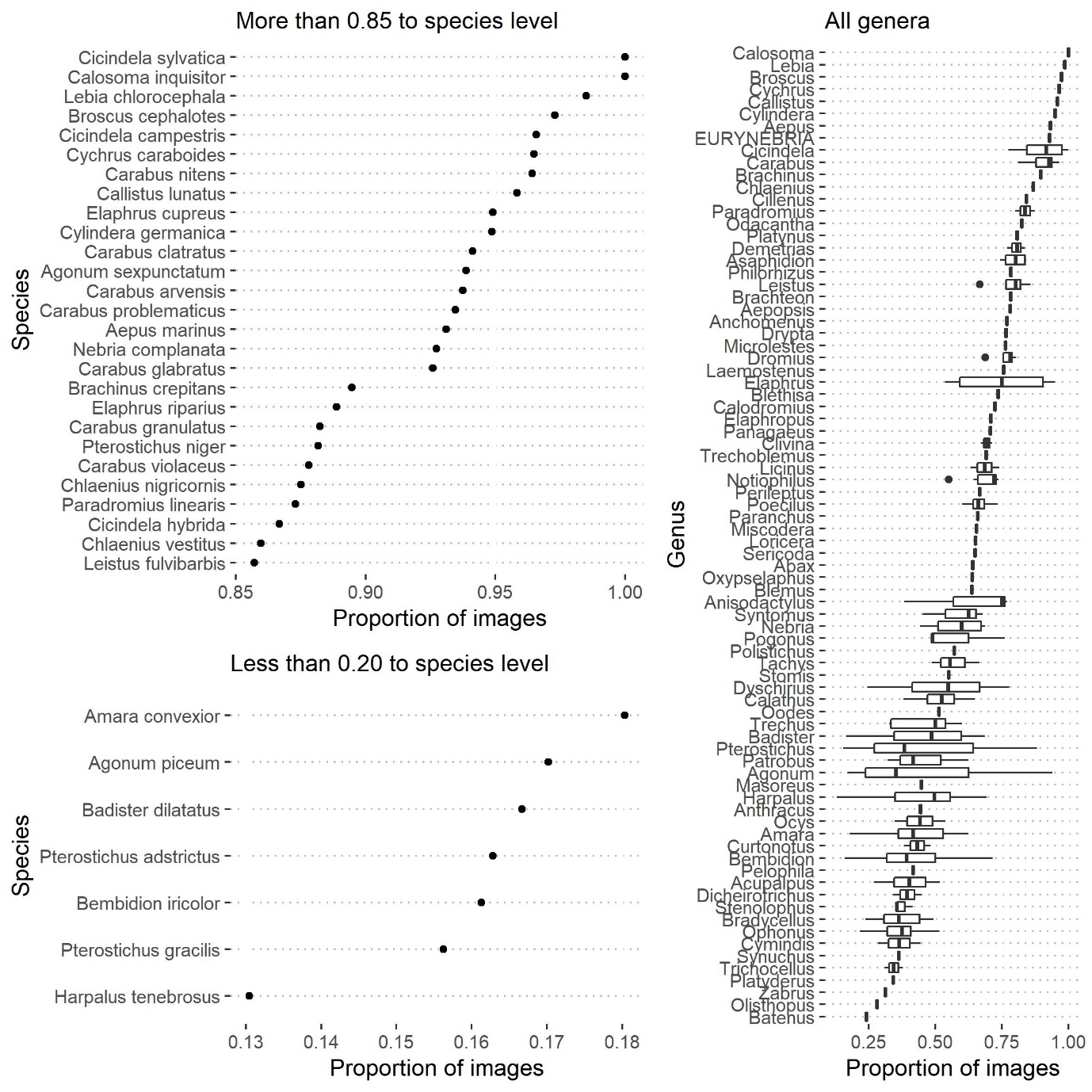


**Figure S3:** Proportion of images classified to species level. Left panel give species with more than 0.85 of images classified at species level or less than 0.20 classified to species level. Right panel give per genera summary of proportion images classified to species level for all species inside each genera.


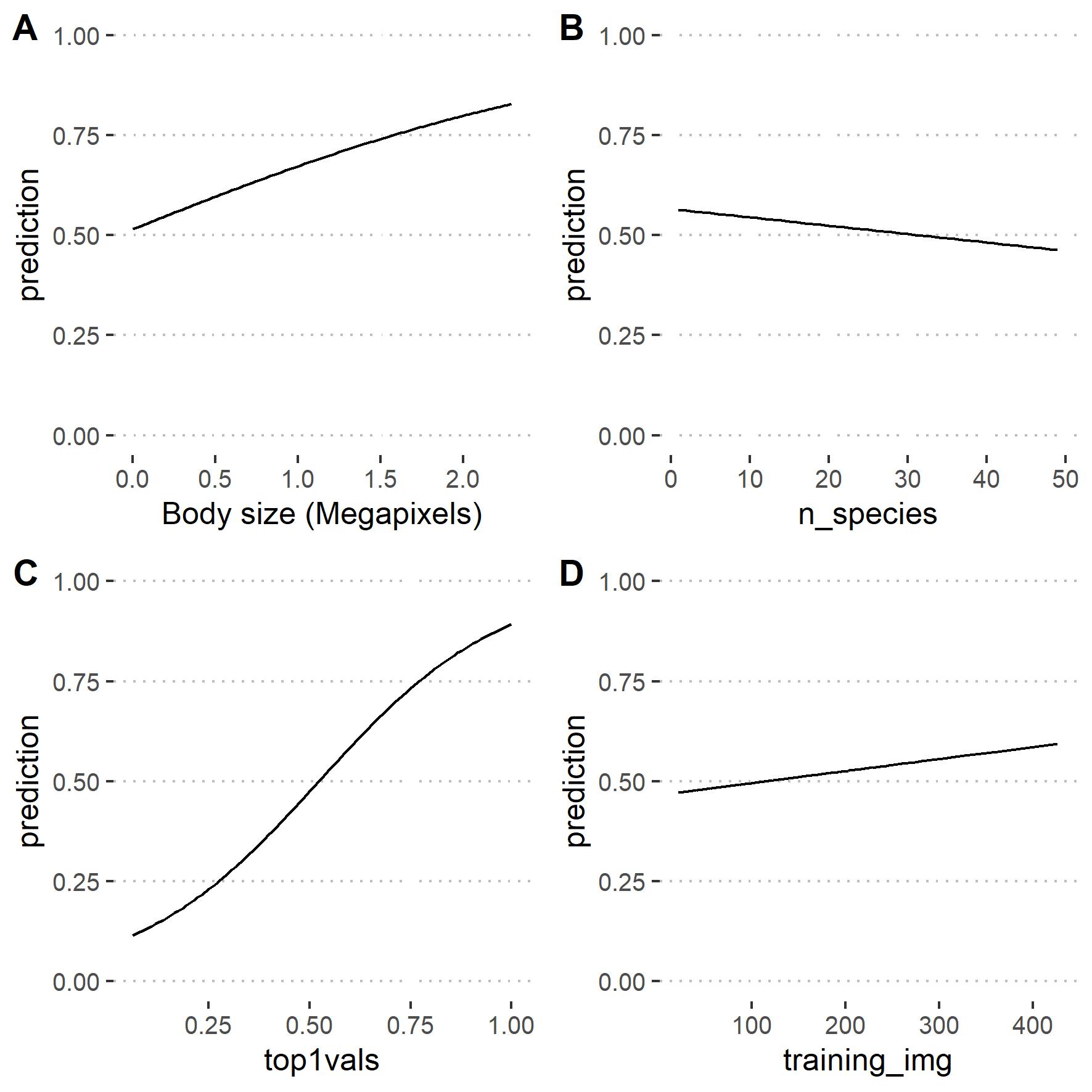


**Figure S4:** Sensitivity analysis of model predictions, within minimum and maximum variation of each variable. All but one variable is constant at mean values for Body size (megapixels) and top1val and median for n_species. A: Change in predicted value for Body size, B: number of species in genus, C: top 1 value in last layer from the convolutional neural network D: number of training images for a species.
